# Resting‐state default mode network connectivity in young individuals with Down syndrome

**DOI:** 10.1002/brb3.1905

**Published:** 2020-11-12

**Authors:** María Dolores Figueroa‐Jimenez, Cristina Cañete‐Massé, María Carbó‐Carreté, Daniel Zarabozo‐Hurtado, Maribel Peró‐Cebollero, José Guadalupe Salazar‐Estrada, Joan Guàrdia‐Olmos

**Affiliations:** ^1^ Department of Health Sciences Centro Universitario de los Valles University of Guadalajara Guadalajara Mexico; ^2^ Department of Social Psychology & Quantitative Psychology Faculty of Psychology University of Barcelona Barcelona Spain; ^3^ UB Institute of Complex Systems University of Barcelona Barcelona Spain; ^4^ Institute of Neuroscience University of Barcelona Barcelona Spain; ^5^ Department of Cognition, Developmental Psychology and Education Faculty of Psychology University of Barcelona Barcelona Spain; ^6^ RIO Group Clinical Laboratory Center for Research in Advanced Functional Neuro‐Diagnosis CINDFA Guadalajara México

**Keywords:** connectivity graphs, default mode network, Down syndrome, fMRI, functional connectivity, intellectual disability, resting state

## Abstract

**Background:**

Down syndrome (DS) is a chromosomal disorder that causes intellectual disability. Few studies have been conducted on functional connectivity using resting‐state fMRI (functional magnetic resonance imaging) signals or more specifically, on the relevant structure and density of the default mode network (DMN). Although data on this issue have been reported in adult DS individuals (age: >45 years), the DMN properties in young DS individuals have not been studied. The aim of this study was to describe the density and structure of the DMN network from fMRI signals in young DS (age: <36 years).

**Method:**

A sample of 22 young people with DS between the ages of 16 and 35 (*M* = 25.5 and *SD* = 5.1) was recruited in various centers for people with intellectual disability (ID). In addition to sociodemographic data, a six‐minute fMRI session was recorded with a 3. T Philips Ingenia scanner. A control group of 22 young people, matched by age and gender, was obtained from the Human Connectome Project (to compare the networks properties between groups).

**Results:**

The values of the 48 ROIs that configured the DMN were obtained, and the connectivity graphs for each subject, the average connectivity graph for each group, the clustering and degree values for each ROI, and the average functional connectivity network were estimated.

**Conclusions:**

A higher density of overactivation was identified in DS group in the ventral, sensorimotor, and visual DMN networks, although within a framework of a wide variability of connectivity patterns in comparison with the control group network. These results extend our understanding of the functional connectivity networks pattern and intrasubject variability in DS.

## Graphical abstract

DMN in Down syndrome. Network indicators in DS. Functional connectivity.

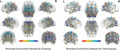

## 1 INTRODUCTION

There is no doubt that neuroimaging studies have instigated a real revolution in the study of cognitive functions. In recent years, works using brain signals (EEG, PET, MRI, or fMRI, among others) have increased considerably and have provided a new way of understanding brain function (Medaglia et al., 2015). There are several reasons for this, although we can fundamentally highlight the technological evolution that allows much more reliable measures of brain functioning and the need to overcome classical paradigms of psychological assessments of cognitive functions.

This type of study has been applied to a multitude of different populations, both at a very basic level and an applied level (Chiesa et al., 2017). Among the latter, studies with populations of special clinical importance have opened a new way of understanding the cognitive functioning associated with certain syndromes or clinical diagnoses and the decline caused by aging. Countless studies can be linked to this idea (Karmiloff‐Smith et al., 2016; Yildirim & Büyükiscan, 2019).

In relation to the different brain signals, the one that has been of most interest in the last ten years is the functional magnetic resonance imaging (fMRI) register. The reason for this preference may be because the fMRI signal allows the generation of representational and mathematical models of brain function, and though it is a somewhat cumbersome record, it is not as invasive as in other signals (Mak et al., 2017). In fact, the increase from the first work in 1994 to date has followed a growing monotonic function (Alegria et al., 2016; Engel et al., 1994; Fox et al., 2015).

However, an exception to this is found in works with populations or samples with intellectual disability (ID), especially when using fMRI signals. In a systematic review, Carbó‐Carreté et al. (2020) identified only 9 papers, of which 7 used the task paradigm and 2 used the resting‐state technique (rs‐fMRI).

Therefore, we must address the low production of fMRI studies in people with ID and more specifically, in people with DS. We believe that the main reason for this situation is because this is a still recent registration technique, applied mostly to populations with a wider occurrence. However, it is gradually being extended to additional populations, such as people with DS. The difficulties associated with this type of recording in this population are centered on (a) the presence of excessive movement during recording, (b) the difficulty in configuring control groups, (c) low IQ levels that prevent the realization of paradigms with more elaborate tasks due to their level of understanding, and (d) according to our experience, the lack of knowledge and misgivings on the part of legal guardians and people with DS themselves (Pujol et al., 2015).

Among all the approaches to study brain connectivity with resting‐state fMRI, the works dedicated to the estimation of the default mode network (DMN) should be highlighted (Anderson et al., 2013; Wilson et al., 2019). As is widely known, the DMN is a network of networks that is generally activated in a resting‐state paradigm. The DMN is an anatomically defined brain area that usually activates when individuals are not centered in any external environment (Buckner et al., 2008). Specifically, it presents high intrinsic activity during resting states without specific task engagement (Beckmann et al., 2005). There is some controversy regarding the act of recording the signal with eyes open or closed. This simple fact has generated various studies to assess the effect of eyes open or closed (Agcaoglu et al., 2019).

The DMN is configured by five networks characterized by the medial prefrontal cortex, medial temporal low structures, posterior cingulate cortex, precuneus, and angular gyrus bilaterally (Spreng & Andrews‐Hanna, 2015). According to Horn et al. (2014), the DMN shows high levels of both functional and structural connectivity and high levels of resting metabolic activity in healthy people (Gusnard & Raichle, 2001). The importance of detecting the DMN is therefore associated with health conditions at rest, and the exceptions to this activation are associated with pathologies with profound cognitive impairment, such as Alzheimer's type dementia (ATD) (Sinai et al., 2016; Yi et al., 2015).

In view of the abovementioned factors, it seems important to establish the functioning of the DMN in people with DS since such a structure, to our knowledge, has only recently been studied by Vega et al. (2015) and Wilson et al. (2019). The first paper analyzes the differences in between‐ and within‐network resting‐state functional connectivity for seven functional networks in DS groups in comparison with TD (typically developing) and WS group (Williams syndrome). The results suggest a global difference in between‐network connectivity in DS group compared with controls across many brain regions. The second paper (Wilson et al., 2019) shows statistically significant differences between a group of people with DS compared to a healthy control group. In particular, the authors point out that the activation of the medial prefrontal cortex is greater in healthy controls and that the opposite effect is present in the middle temporal gyrus network, in which the activation in DS individuals is somewhat greater than that in healthy controls.

It is important to clarify that when we speak of increased activation in a specific area of the brain, we mean that the signal values are higher in that area compared to the other group. It is obviously a neuronal activation effect that the fMRI signal detects. This last work (Wilson et al., 2019) is especially important since it analyzed a sample of DS people in the absence of brain beta‐amyloid and, therefore, free of the cognitive impairment associated with ATD. However, in samples of people with DS, cognitive impairment is still present because of intellectual disability.

In fact, both works were done with adults (age range: 30–55 years), and there is no evidence obtained in younger people. As these are adults with ATD, the covert diagnostic effect of ATD‐associated DMN alteration cannot be avoided (Rubenstein et al., 2020). This effect is avoided when evaluating younger DS persons. Thus, studies of the DMN in younger DS individuals will allow analysis of the network with fewer effects not directly associated with DS.

The main objective of this study was to estimate the functional connectivity network based on the DMN in a resting state in young people diagnosed with DS in comparison with the brain connectivity network in a group of healthy individuals with no DS. Second, we propose to estimate different indicators to explore and describe the behavior of the pattern of functional connectivity networks in the DS group and control group.

## 2 MATERIALS AND METHODS

### 2.1 Participants

The initial sample was composed of a total of 35 persons with DS between 16 and 35 years of age (*M* = 24.7 and *SD* = 5.5), and 26.5% were women (*n* = 9). The sampling was opportunistic, and recruitment took place through contact with different associations dedicated to DS in the state of Jalisco (México) (63.6% of participants) and in Spain (36.4%). The inclusion criteria were as follows: (a) age between 16 and 35 years and (b) formal diagnosis of DS including evidence of karyotyping results. The exclusion criteria were as follows (a) evidence of other comorbid diagnoses implying cognitive dysfunction, (b) inability to obtain consent from legal caregivers, (c) the presence of medication affecting cognitive functions, and (d) the presence of translocation or mosaicism.

After recording the fMRI signal, 10 of the subjects were eliminated due to excessive movement during the recording, and some of them were even removed for the same reason after having repeated the recording. Records with movement greater than ± 2 degrees (or greater than half voxel size) were eliminated and not included in this paper analysis and therefore not statistically analyzed. Thus, the final sample for which fMRI was analyzed was composed of a total of 22 persons with DS, with the following observed age distribution: *M* = 25.5 and *SD* = 5.1. The distribution of the final sample consisted of 8 people from Mexico and 14 from Barcelona, the average age was *M* = 25.6 (*SD* = 5.2), and 22.7% were women (*n* = 5). In relation to the degree of severity, it revealed that 4.5% had a limited intellectual disability, 36.4% had mild intellectual disability, 40.9% had moderate intellectual disability, and finally, 18.2% had profound intellectual disability. This classification appears in the official report that each DS person presented at the time of incorporation into the study and limited intellectual disability is connected with the borderline zone so this category does not appear in ICD‐10 categories (Codes F70‐F79). All persons of the DS group were right‐handed.

A control group (*n* = 22) was included to compare the indicators of complex networks analyzed in DS population. These subjects were obtained from the Human Connectome Project (http://www.humanconnectomeproject.org/), specifically from the open‐access dataset Autism Brain Imaging Data Exchange I (ABIDE I). The ABIDE I is an image repository comprised of 17 international sites and collect structural and rest fMRI scans from people with Autism spectrum disorder and healthy control groups. All data, including the phenotypic datasets and the protocol of acquisition parameters, are available in http:// http://fcon_1000.projects.nitrc.org/indi/abide/abide_I. Only the control group of ABIDE I dataset was used, and the subjects were selected to be matched with DS sample by chronological age (*M* = 24,68; *SD* = 4.90; maximum 2 years of difference in some subjects) and gender (22.7% were women). No statistical differences were found in relation to age (*t* = 0.568; *df* = 42; *p*_bilateral_ = .573).

### 2.2 Instruments

The data from this work, only for DS group, are part of a larger protocol in which the relationship between the brain signal (fMRI) and various variables connected with cognitive performance, quality of life, and physical activity are studied in DS population. In this case, for the following study, only some instruments were used to check that the inclusion criteria were met. In all cases, the following assessment and measurement elements were administered:

1. The Dementia Screening Questionnaire for Individuals with Intellectual Disabilities (DSQIID) has an internal consistency estimated with α by a Cronbach value of 0.91 (Deb et al., 2007). It was used to rule out signs of dementia.
2. Ad hoc questionnaires were used to assess the clinical and educational history, and the following variables were collected: age, sex, place of residence, and degree of intellectual disability. This questionnaire can be used for research purposes only and can be obtained by requesting it from the authors.

### 2.3 Procedure

For the DS group, informed consent was obtained from each participant prior to the first neuropsychological screening session in accordance with the Declaration of Helsinki. All the phases of the protocol were approved by the ethics committee of the Bioethics Committee of the University of Barcelona (Spain) and the University of Guadalajara (México). In accordance with this document, informed consent was obtained from the parents of each person with DS and from the persons with DS themselves. In the case of DS people, our protocol included a part in which the tasks that we would perform were explained in detail to each person and confirmation of the understanding of this part by the person with DS was required. In addition, a medical report was obtained for each participant to rule out incompatibilities with the scanner register. All participants were evaluated in two registration sessions by previously trained researchers. The administration sequence was the same for all subjects, and the scales referenced above were administered first to avoid fatigue bias. All questionnaires were heteroadministered. The DSQIID scale was completed by the parents of the people with DS, while the sociodemographic record was obtained from the people with DS, and all the assessments were administered during the same day. Data were collected from March 2018 to July 2019.

### 2.4 MRI image acquisition and preprocessing

After the administration of the scales, the participants underwent the fMRI recording sequence in the following order: T1‐weighted, T2‐weighted, FLAIR, and 6‐min resting state. Two system models 3 T Philips Ingenia scanner (Phillips Healthcare) were used (one located at the Clinical Laboratory, Integral Medical Diagnostic Center of Guadalajara's RIO Group Center in Jalisco, and the other at the Pasqual Maragall Foundation in Barcelona). A T1‐weighted turbo field echo (TFE) structural image was obtained for each subject with a 3‐dimensional protocol (repetition time [TR] = 2,300 ms, echo time [TE] = 2,980 ms, 240 slices, and field of view [FOV] = 240 × 240 × 170). The image acquisition was in the sagittal plane. For the functional images, a T2*‐weighted (BOLD) image was obtained (TR = 2000 ms, TE = 30 ms, FOV = 230 × 230 × 160, voxel size = 3 × 3 × 3 mm, 29 slices). The image acquisition was in the transverse plane. During scanning, the participants were instructed to relax, remain awake, and keep their eyes open and fixed on a cross symbol on the screen.

The structural imaging data were analyzed using an FSL (http://www.fmrib.ox.ac.uk/fsl/, RRID:SCR_002823) preprocessing pipeline adapted under authorization from Diez et al. (2015), with its parameters adjusted to fit our experimental data, including a motion correction procedure to solve the undesired head movements in the fMRI sessions. T1‐weighted images were reoriented to match the same axes as the templates, and a resampled AC‐PC aligned image with six degrees of freedom (*df*) was created. All nonbrain tissue was removed to obtain an anatomical brain mask that would be used to parcel and segment the T1‐weighted image data. The use of DARTEL templates was ruled out since some previous analyses did not identify significant differences in relation to the use of general templates (Jacola et al., 2011). The final step involved registering our structural imaging data to normalized space using the Montreal Neurological Institute reference brain based on the Talairach and Tournoux coordinate system (Ashburner & Friston, 1999). Finally, during the fMRI recording, a caregiver of the DS person evaluated was present inside the scanner room, dedicated to their care to reassure the DS persons, and thus avoid unnecessary movements or aberrant behaviors or lack of adherence to rejection instructions. He was only present in the room without interacting with the person evaluated, but we found that the mere presence of caregivers or parents greatly reduced aberrant movement or distractions.

Regarding the control group, the acquisition was performed in different institutions of the United States. As in the case of the DS group, all the participants performed fMRI recording sequence: T1, T2, FLAIR, and between 6‐ and 9‐min resting state. The repetition time (TR) in all cases was 2,000 ms, and the voxel size was different for every protocol. Moreover, due to the extra minutes in resting, in all the cases in the control group, the number of volumes was greater than in the DS group (oscillating between 240 volumes and 300). Therefore, we used only the first 220 volumes corresponding to the ones used in the DS group.

### 2.5 Regions of interest

For both groups, the automated anatomical labeling (AAL) atlas (Tzourio‐Mazoyer et al., 2002) was used to define the regions of interest (ROIs). This atlas contains 45 cortical and subcortical areas in each hemisphere (90 areas in total and available by request). To acquire the full signal of a given ROI, it is necessary to compute an average over the entire time series of all the voxels of a given brain area following the AAL atlas. In relation to the objective of the present study of the brain connectivity patterns in Down syndrome, we select only regions involved in the DMN network. These regions were divided into anterior, ventral, and posterior subnetworks based on the classification proposed by Huang et al. (2015). The anterior DMN (DMNa) subnetwork included the anterior cingulate, paracingulate gyrus, insular cortex, and frontal and temporal poles. The ventral DMN (DMNv) subnetwork included the precuneus and middle cingulate, hippocampus, and parahippocampal gyrus. The posterior DMN (identified as simple DMN) subnetwork included the lateral parietal and middle temporal gyrus. Additionally, the sensorimotor (SM) has been included, which consists of the frontal lobe; the precentral, midfrontal, and supramotor areas; and the postcentral, supramarginal, and paracentral areas. Likewise, the visual network (V) is composed of the primary visual cortex, the calcarine fissure, the cuneus, the occipitotemporal gyrus, and the occipital lobe (Farras‐Permanyer et al., 2019). Table 1 shows the entire ROI list.

**TABLE 1:** Relationship of ROIs for the construction of the DMN and subnetworks considered according to the AAL90 atlas

DMN		DMN anterior		DMN ventral		Sensorimotor		Visual	
Roi	Region name	Roi	Region name	Roi	Region name	Roi	Region name	Roi	Region name
59	Parietal_Sup_L	29	Insula_L	35	Cingulum_Post_L	1	Precentral_L	43	Calcarine_L
60	Parietal_Sup_R	30	Insula_R	36	Cingulum_Post_R	2	Precentral_R	44	Calcarine_R
61	Parietal_Inf_L	31	Cingulum_Ant_L	37	Hippocampus_L	7	Frontal_Mid_L	45	Cuneus_L
62	Parietal_Inf_R	32	Cingulum_Ant_R	38	Hippocampus_R	8	Frontal_Mid_R	46	Cuneus_R
85	Temporal_Mid_L	87	Temporal_Pole_Mid_L	39	Parahippocampal_L	19	Supp_Motor_Area_L	47	Lingual_L
86	Temporal_Mid_R	88	Temporal_Pole_Mid_R	40	Parahippocampal_R	20	Supp_Motor_Area_R	48	Lingual_R
				55	Fusiform_L	57	Postcentral_L	49	Occipital_Sup_L
				56	Fusiform_R	58	Postcentral_R	50	Occipital_Sup_R
				65	Angular_L	63	Supramarginal_L	51	Occipital_Mid_L
				66	Angular_R	64	Supramarginal_R	52	Occipital_Mid_R
				67	Precuneus_L	69	Paracentral_Lobule_L	53	Occipital_Inf_L
				68	Precuneus_R	70	Paracentral_Lobule_R	54	Occipital_Inf_R

### 2.6 Statistical analysis

Once the images were preprocessed, correlation matrices were obtained between the 48 ROIs for each subject evaluated and group. To avoid the aberrant effect of values in some especially high or low ROIs (outliers), the jackknife correlation was estimated. There are other simulation possibilities in estimating statistical significance, but for small samples it is still recommended. This technique consists of calculating all the correlation coefficients between all the possible ROI pairs if one of the observations is excluded on each occasion. The average of all the correlations for each ROI pair attenuates the effects of the outliers. Each jackknife correlation coefficient is estimated using the following expression:

$$\theta_{\left(\text{ROI}i\text{,ROI}j\right)}=\text{Jackknife}\;\text{Correlation}\;\text{Mean}\left(\text{ROI}i,\text{ROI}j\right)=\frac{1}{n}\sum_{k=1}^{n}r_{i}$$

where *r_i_* is Pearson's correlation between each pair of ROIs and n is the sample number in which the correlations in each pair have been estimated by extracting the record (volume) *i*. The *SE* of each average was also estimated from the expression:

$$SE=\sqrt{\frac{n‐1}{n}\sum_{i=1}^{n}{\left(r_{i}‐\theta \right)}^{2}}$$

This allows the estimation of confidence intervals for each correlation coefficient. Selecting between the correlation coefficient obtained with the whole sample or the one obtained through jackknife estimation depends on the bias value obtained. The bias is defined by the following expression:

$$\text{Bias}=\left(n‐1\right)\times \left(\theta ‐\hat{r}\right).$$

For each correlation between ROIs, the value of bias was obtained, and when this was close to 0, the average jackknife value was used. In cases where bias was different from 0, the value of the lower limit of the confidence interval was used to avoid the probability of a type I error. The use of the value 0 as a reference point is justified in view of the previous expression. For there to be no sense, the difference $\left(\theta ‐\hat{r}\right)$ must be close to 0. This would indicate the absence of sampling error and, therefore, the best possible estimate. To perform these analyses, the dist R library (3.6.3) was used, and all correlations were positivized. Once the matrices were configured for each person with DS, the global matrix was generated for the entire sample using the stacked raw data. All the correlation matrices thus estimated were transformed to *Z*‐scores by means of the Fisher transformation to facilitate the variance‐stabilizing transformation:

$$z=\frac{1}{2}\ln\left(\frac{1+r}{1‐r}\right).$$

All the two matrices were binarized using degrees of significance lower than *p* < .001 were considered significant to further reduce type I errors. The Z matrices were used as a main matrix to estimate distance between ROIs and the binarized matrices were used as an adjacent matrices to estimate each network. To further analyze the density of the functional connectivity networks for each participant and for the entire sample, we studied the structures that arose in the whole‐brain analysis, including only the DMN areas described in abovementioned Table 1.

Graph plots from each *z_i_* correlation matrix were built through the qgraph package for R (Epskamp et al., 2012). The results and maps obtained from these results were displayed by BrainNet Viewer (Xia et al., 2013). Database is available upon request to the authors.

## 3 RESULTS

**FIGURE 1:**
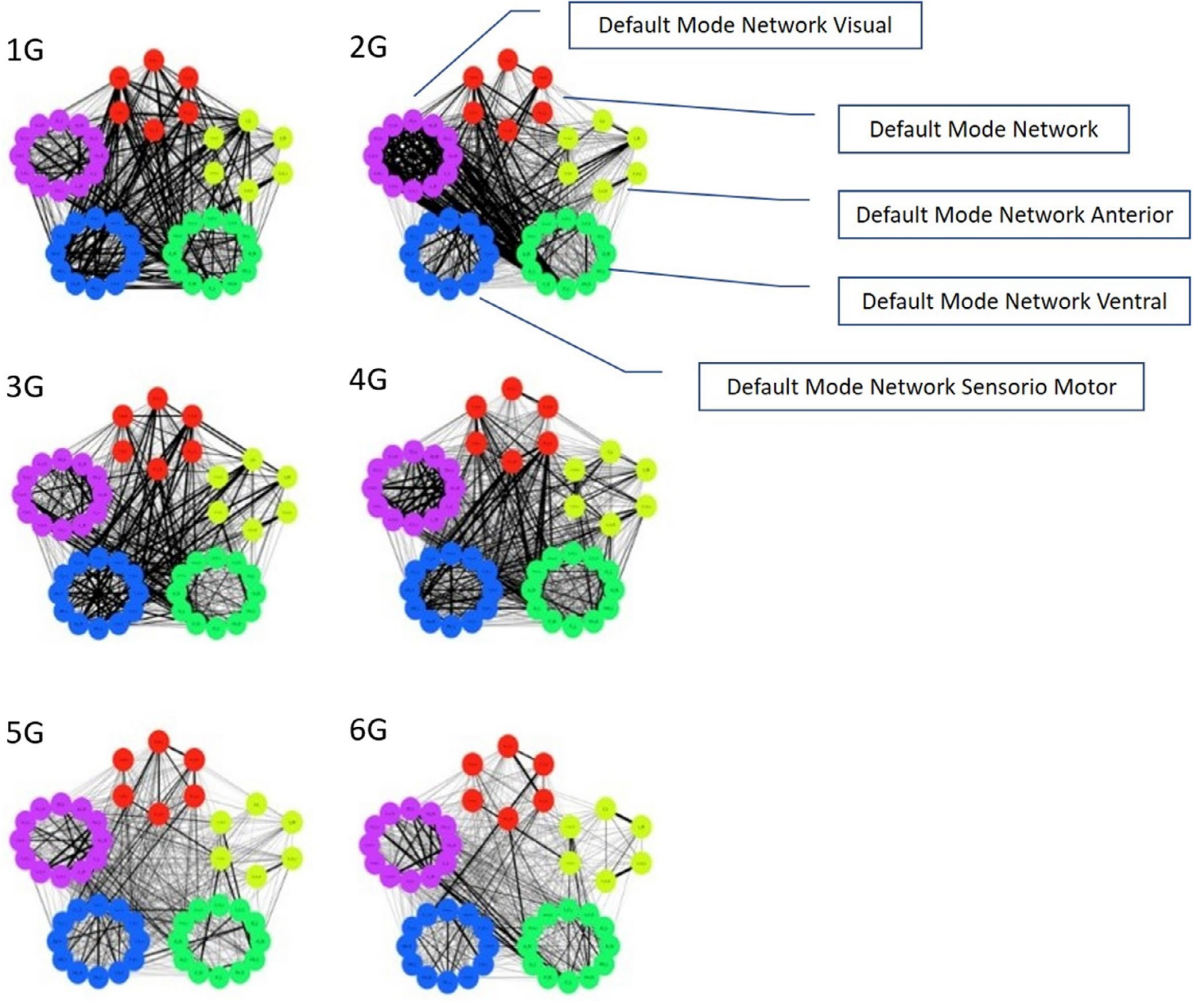
Representative graphs of high connectivity (1g and 2g); medium connectivity (3g and 4g); and low connectivity (5g and 6g). Red: DMN posterior; yellow: DMN anterior; green: DMN ventral; blue: sensorimotor; and purple: visual. The number of each ROI is listed in Table 1

Figure 1 shows a representation of the functional connectivity network of six of the 22 DS subjects. The simple visual inspection of the networks shows a very variable behavior, and a certain continuum can be identified in terms of the density of the connectivity network. We selected some of the characteristic networks of subjects with high (1G and 2G) connectivity in the global DMN, of those with medium connectivity (3G and 4G), and finally of those with low connectivity intensity (5G and 6G). They were obtained from the values of correlation coefficients transformed to Fisher's *z_i_* values, establishing a threshold of *p* < .001. Figure 1 shows the networks of these selected subjects.

**FIGURE 2:**
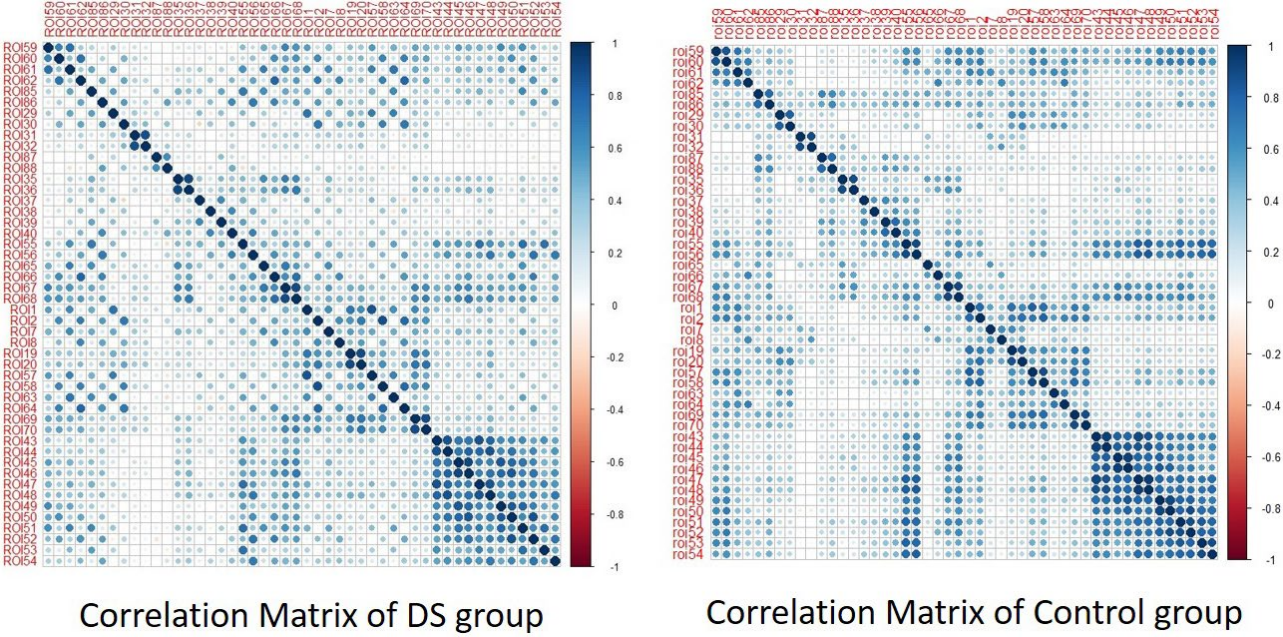
Average correlation matrix correlogram for each group

**FIGURE 3:**
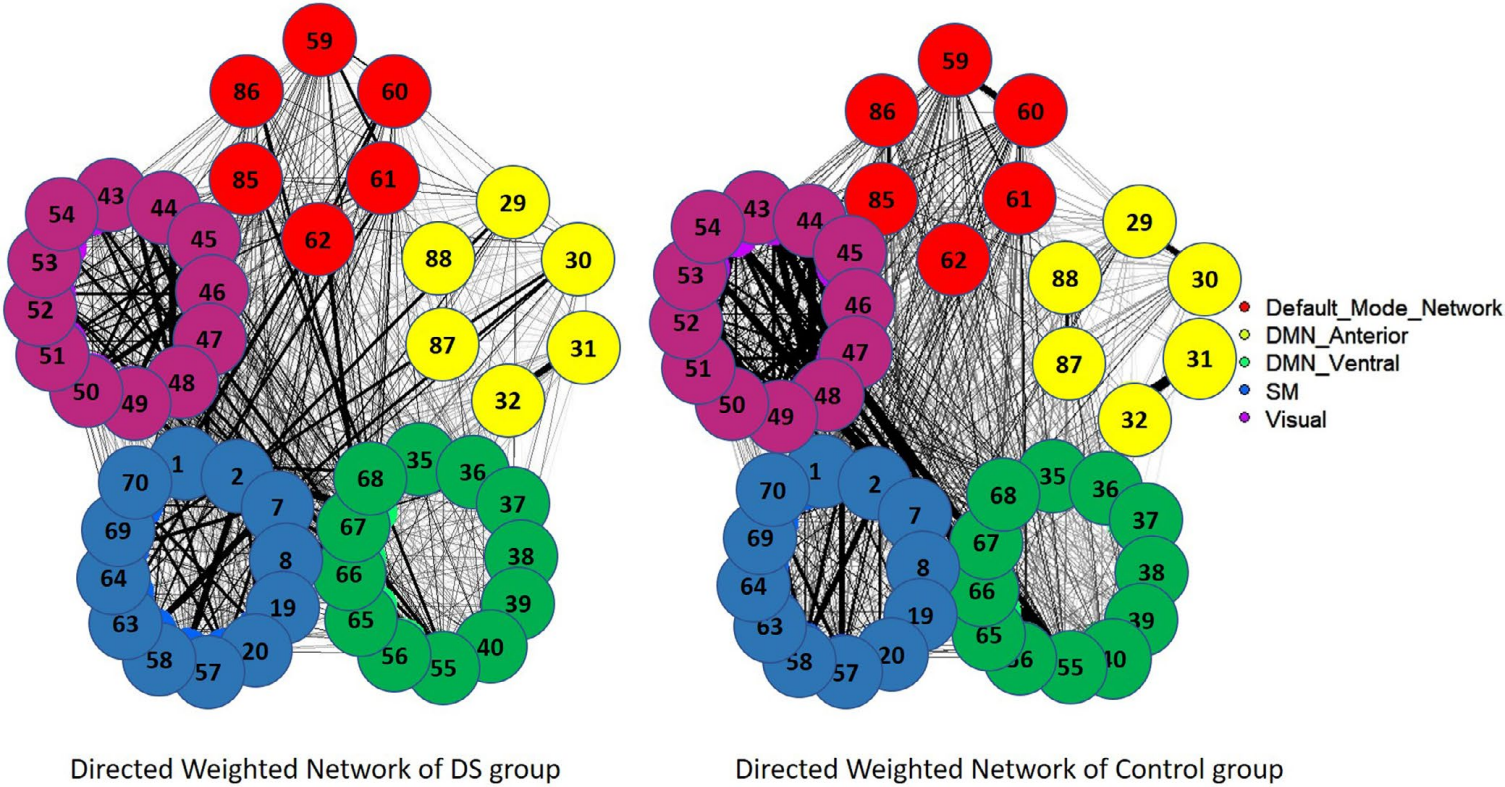
Average graph of functional connectivity on the DMN network for people with DS, estimated from Fisher's transformed *z_i_* values. Red: DMN posterior; yellow: DMN anterior; green: DMN ventral; blue: sensorimotor; and purple: visual. The number of each ROI is listed in Table 1

The simple graphical inspection of Figure 1 indicates a wide variability in the connectivity density of the DMN network in the sample of DS persons, and they did not show a regular pattern regarding the connectivity density. It was not necessary to reproduce this analysis in the control group, since the evidence of variability in the directed networks in the DS group was sufficient to try to study both groups using the average correlation matrix. According to this situation, we chose to show the analyses performed with the average correlation matrix representative for each group. To do so, Figure 2 shows the correlogram between the 48 ROIs constituting all DMN networks (corrplot library of R), and Figure 3 shows the average connectivity network established from the average correlation matrix (qplot library of R).

Figure 2 indicates very moderate average connectivity levels in both groups except in some subnetworks (such as the visual network). The visual inspection of the correlograms shows a higher similarity between global correlation values. As previously stated, Figure 3 shows these effects more clearly through the estimation of the corresponding directed network for each group.

**FIGURE 4:**
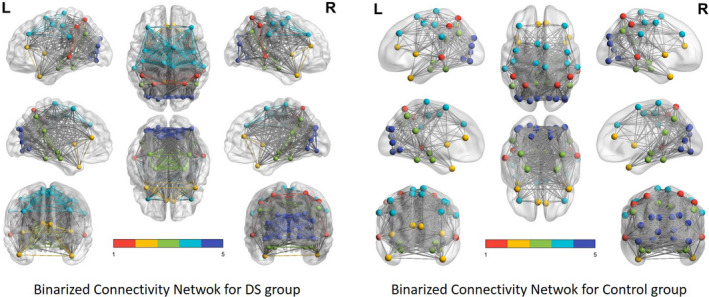
DMN network of binary functional connectivity in people with DS. Red: DMN posterior; yellow: DMN anterior; green: DMN ventral; blue: sensorimotor; and purple: visual

From these values, the binary functional connectivity matrix for each group was obtained, and the results were presented using BrainNet Viewer. Figure 4 shows the results obtained after this procedure.

**FIGURE 5:**
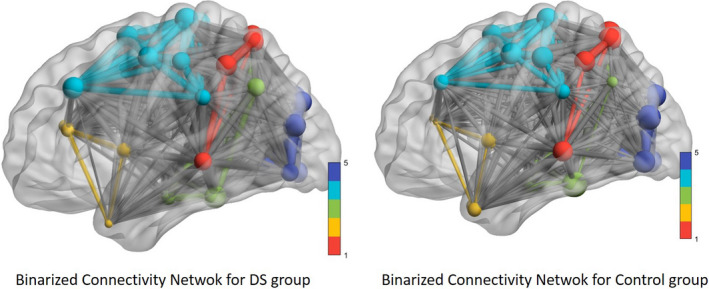
DMN network of heavy functional connectivity in people with DS. Red: DMN posterior; yellow: DMN anterior; green: DMN ventral; blue: sensorimotor; and purple: visual. The size of the ROI is proportional to the connectivity that present, and the edges between ROIs are proportional to the *z_i_* transformation between them

The graphical representation of the functional connectivity network was also obtained from the heavy matrix and using the degree of each ROI to establish its connection level with the rest of the ROIs. Figure 5 shows these networks for both groups.

Finally, to provide the data corresponding to the importance of each DMN ROI in relation to network connectivity, we present in Table 2 the path length values for each ROI estimated from the weighted global matrix for each group and ordered from highest to lowest connectivity with the rest of the regions in the DS group to facilitate the interpretation.

**TABLE 2:** Degree (weighted) for each ROI, ordered from the highest value to the lowest in DS group

Subnetwork	ROI Number AAL90 atlas	Description	Degree	
			DS Group	Control Group
DMNv	68	Precuneus_R	23.21	10.64
	67	Precuneus_L	22.56	11.17
Visual	48	Lingual_R	22.34	9.74
	46	Cuneus_R	21.06	8.16
	47	Lingual_L	20.93	9.33
	45	Cuneus_L	20.25	10.58
SM	69	Paracentral_Lobule_L	20.22	7.53
	70	Paracentral_Lobule_R	20.01	7.59
Visual	44	Calcarine_R	19.47	3.78
	51	Occipital_Mid_L	19.39	3.85
	50	Occipital_Sup_R	19.16	6.72
DMNv	66	Angular_R	18.92	7.82
SM	19	Supp_Motor_Area_L	18.67	5.87
DMNv	55	Fusiform_L	18.66	5.97
Visual	52	Occipital_Mid_R	18.65	6.57
SM	20	Supp_Motor_Area_R	18.64	6.88
Visual	43	Calcarine_L	18.63	8.16
	54	Occipital_Inf_R	18.37	9.05
DMNv	56	Fusiform_R	18.22	11.59
SM	7	Frontal_Mid_L	18.13	11.46
DMN	61	Parietal_Inf_L	17.80	5.54
Visual	49	Occipital_Sup_L	17.78	7.48
DMN	62	Parietal_Inf_R	17.75	10.97
	59	Parietal_Sup_L	17.70	11.49
SM	1	Precentral_L	17.68	10.12
DMN	60	Parietal_Sup_R	17.46	11.06
DMNv	36	Cingulum_Post_R	16.78	7.32
SM	2	Precentral_R	16.66	6.87
	8	Frontal_Mid_R	16.35	9.43
DMNv	35	Cingulum_Post_L	15.89	9.01
DMN	86	Temporal_Mid_R	15.52	9.99
DMNv	65	Angular_L	15.36	10.27
SM	57	Postcentral_L	15.31	7.19
	58	Postcentral_R	15.30	7.89
DMNv	40	Parahippocampal_R	15.13	10.01
DMN	85	Temporal_Mid_L	14.88	9.37
Visual	53	Occipital_Inf_L	14.86	9.97
SM	64	Supramarginal_R	14.74	9.63
	63	Supramarginal_L	13.07	10.01
DMNa	29	Insula_L	12.10	9.86
DMNv	39	Parahippocampal_L	11.76	11.33
DMNa	30	Insula_R	11.74	11.54
	31	Cingulum_Ant_L	11.74	10.41
	32	Cingulum_Ant_R	10.24	9.76
DMNv	38	Hippocampus_R	8.86	11.40
	37	Hippocampus_L	8.77	10.23
DMNa	87	Temporal_Pole_Mid_L	6.03	10.31
	88	Temporal_Pole_Mid_R	5.77	10.26

Abbreviations: DMN, posterior; DMNa, anterior; DMNv, ventral; SM, Sensorimotor.

In addition, we carried out a secondary analysis to assess whether the origin of the samples from Mexico or Spain could have generated some type of bias. No statistically significant result was obtained indicating the absence of differences between subsamples. We used nonparametric test to avoid the heteroscedasticity between groups and small sample. The significance was between 0.432 and 0.876 in Mann–Whitney test.

## 4 DISCUSSION

The results obtained in the analysis of the functional connectivity networks of the DMN networks in people with DS have shown a wide variability in the density of connectivity that each participant presents and an average result in which greater intranetwork connectivity is shown in the motor–sensory network and in the visual network. The rest of the connections between ROIs are statistically significant but of lesser intensity. The study of the networks and the degree of each ROI indicate that, in fact, the motor sensor network and the visual network present higher values of connection intensity, while the DMNa and DMNv networks present lower intensity of connections than the rest, although the latter are presented in a very disaggregated way. The connectivity network for the control group indicates a similar network to that described in the case of the DS group but with lower intensity connection values (edges) between ROIs.

First, our results show that the intensity of functional connections between the ROIs that make up the DMN and between the subnetworks that can be identified are extremely variable and present in a certain continuum and that especially in our sample, there is no generalized interruption of the DMN, as was reported by Wilson et al. (2019) in older people with DS. The individualized graphs indicate such a level of variability that the graphs and average results should be analyzed with caution. This effect is not different from that reported in similar studies in other populations (Smitha et al., 2017), including networks estimation in healthy people (Farras‐Permanyer et al., 2019).

More interesting is the average network estimate obtained from the average correlation matrix. In this case, the result indicates that as mentioned previously, the DMN of people with DS is characterized by greater connectivity in the DMNv network related to visuospatial processing and the coding of information through the visual and auditory pathways, as well as the sensorimotor and visual areas since they have the highest degree within the ROIs as opposed to the DMNa in charge of emotional processing, mood control, and the subsequent DMN network related to information recognition. The control group shows the same connections found for the DS group, as expected, but with lower intensity levels than the DS group.

This is consistent, to some extent, with the work of Pujol et al. (2015), which refers to greater connectivity in the ventral brain system as opposed to the anterior brain system. Therefore, our results indicate a profile of connectivity in young people with DS that, unlike the profile of young people without DS, reports less functional connectivity and/or correlative strength within the DMN between the medial prefrontal cortex, that is, the DMNa, and the posterior cingulate cortex, also known as the DMNv, which progressively decreases as age increases. This is congruent with what is reported in the same line by Mak et al. (2017).

People with DS showed somewhat different functional connectivity networks than expected in the DMN according to the average graph in comparison with the control group. They showed greater self‐referential mental activity based on the strength of the association obtained in the DMNv, although in a somewhat fragmented way, as did the processing of the visual area and the control of involuntary movements of the sensorimotor area in a resting state. During the recognition of spatially oriented stimuli, the system that understands speech located in a DMN subarea and social and emotional association processing carried out by the DMNa showed little functional connection strength.

We should, however, look for some reasonable explanation for the overactivation of the sensory–motor and visual areas. It seems plausible to assume that the people with DS evaluated moved their upper and lower extremities and, in addition, had abnormalities with eye tracking during recording, which could generate these differences. Similarly, given the morphological characteristics of people with DS such as decreases in the cerebellum, prefrontal cortex, hippocampus, and circumvolution of the temporal lobe and with networks and circuits exhibiting less extension and a lower organizational capacity (Flórez et al., 2015), it may be complementary to the previous description to explain the overactivation detected in the average graph; this presents greater connectivity in the aforementioned subnetworks, little symmetry between subnetworks, and low intensity of functional association between the DMN subnetworks.

This work presents some limitations to consider. First, the sample size is limited, the control group is drawn from general databases, and therefore, they have not been recruited in the strict sense, and DSQIID was not studied for its psychometric properties for its Spanish version. The inclusion of a control group, being a relevant contribution, does not stop presenting difficulties in interpreting the results due to differences in brain morphology between groups. Despite this, it has been included to better establish network properties in the DS group. Finally, it should be noted that this work should be complemented with an exhaustive analysis of the cognitive functions of DS people to assess possible links of the properties of the connectivity network with the distributions of the cognitive performance tests.

The results obtained and their interpretation lead us to conclude that the DMN network in the DS population can be affected by the difficulty of recording with interfered movements (motor and visual) and a clear asymmetry between subnetworks. Obviously, the lack of this type of effects in the control group must be attributed to the absence of alterations in the network, both in cerebral and behavioral terms, in non‐DS people.

Likewise, we can establish that there is clear intrasubject variability that shows very different behavior in terms of the density of connectivity detected in the participants. This behavior is not exclusive to the sample used in this study since the variability in the non‐DS populations is similar. However, it seems necessary to further study density‐modifying variables that can explain part of the observed variation. It will be necessary to study whether the effect of more neurostructural mechanisms or the effect of external variables (e.g., cognitive factors including the level of cognitive response or possible cognitive reserve effects or more psychological aspects such as quality of life) could explain this phenomenon. Complementarily, the relationship that DS has, for example, with organically based health conditions such as hypothyroidism and congenital heart disease, as well as with the use of medications, should be investigated. Similarly, longitudinal studies could allow to analyze the network properties of the DMN network from the follow‐up of young to elderly subjects and to evaluate the possibility that this network becomes an early biomarker of ATD.

## CONFLICTS OF INTEREST

The authors claim to have no conflict of interest in this study.

## AUTHOR CONTRIBUTION

María Dolores Figueroa‐Jimenez and Cristina Cañete‐Massé collected the data, performed statistical analysis, and wrote the paper. María Carbó‐Carreté and Maribel Peró‐Cebollero collected the data and wrote the paper. Daniel Zarabozo‐Hurtado collected the data. José Guadalupe Salazar‐Estrada supervised the study. Joan Guàrdia‐Olmos collected the data, performed statistical analysis, wrote the paper, and supervised the study.

### Peer Review

The peer review history for this article is available at https://publons.com/publon/10.1002/brb3.1905.

## Data Availability

Database is available upon request to the authors.
